# Breaking bad news: doctors’ skills in communicating with patients: Dando más notícias: a habilidade dos médicos em se comunicar com os pacientes

**DOI:** 10.1590/1516-3180.20160221270117

**Published:** 2017-05-29

**Authors:** Francisco José Ferreira da Silveira, Camila Carvalho Botelho, Carolina Cirino Valadão

**Affiliations:** I MD, MSc, PhD. Assistant Professor, Department of Public Health, Faculdade de Ciências Médicas de Minas Gerais (FCMMG), Belo Horizonte (MG), Brazil.; II MD. Psychiatry Medical Residency, Santa Casa de Misericórdia de São Paulo, São Paulo (SP), Brazil.; III MD. General Clinics Medical Residency, Risoleta Neves Hospital, Belo Horizonte (MG), Brazil.

**Keywords:** Truth disclosure, Professional-patient relations, Health communication, Teach-Back communication, Models, educational

## Abstract

**CONTEXT AND OBJECTIVE:**

Breaking bad news is one of doctors’ duties and it requires them to have some skills, given that this situation is difficult and distressful for patients and their families. Moreover, it is also an uncomfortable condition for doctors. The aim of this study was to evaluate doctors’ capacity to break bad news, ascertain which specialties are best prepared for doing this and assess the importance of including this topic within undergraduate courses.

**DESIGN AND SETTING:**

Observational cross-sectional quantitative study conducted at a university hospital in Belo Horizonte (MG), Brazil.

**METHODS:**

This study used a questionnaire based on the SPIKES protocol, which was answered by 121 doctors at this university hospital. This questionnaire investigated their attitudes, posture, behavior and fears relating to breaking bad news.

**RESULTS:**

The majority of the doctors did not have problems regarding the concept of bad news. Nevertheless, their abilities diverged depending on the stage of the protocol and on their specialty and length of time since graduation. Generally, doctors who had graduated more than ten years before this survey felt more comfortable and confident, and thus transmitted the bad news in a better conducted manner.

**CONCLUSION:**

Much needs to be improved regarding this technique. Therefore, inclusion of this topic in undergraduate courses is necessary and proposals should be put forward and verified.

## RESUMO

**CONTEXTO E OBJETIVO:**

Dar más notícias, além de dever do médico, requer certas habilidades de sua parte, por se tratar de situação difícil e angustiante para o paciente e seus familiares, assim como desconfortável para os profissionais da saúde. O objetivo deste estudo é avaliar a capacidade dos médicos em dar más notícias, assim como as especialidades mais preparadas e a importância da inclusão do tema para a graduação.

**TIPO DE ESTUDO E LOCAL:**

Estudo observacional, transversal, quantitativo, realizado em hospital universitário de Belo Horizonte, Minas Gerais, Brasil.

**MÉTODOS:**

Este estudo utilizou de questionário baseado no protocolo SPIKES que foi respondido por 121 médicos deste hospital universitário. O questionário investigou suas atitudes, posturas, modos e medos em relação a dar más notícias.

**RESULTADOS:**

A maioria dos médicos não teve problemas quanto ao conceito de más notícias, contudo, as habilidades divergiram dependendo da etapa do protocolo, assim como quanto a especialidade e tempo de formado. De modo geral, os médicos formados há mais de 10 anos se sentem mais confortáveis e confiantes, e transmitem tal informação de maneira mais bem conduzida.

**CONCLUSÃO:**

Muito se tem a aprimorar em relação a essa técnica. Desse modo, a inclusão do tema durante a graduação é necessária e propostas devem ser sugeridas e averiguadas.

## INTRODUCTION

The latest edition of the medical ethics code (2010: in chapter V, article 34) in Brazil states that doctors are forbidden from not telling the truth to patients about their diagnosis, prognosis, treatment risks and treatment goals. The only exception is information that could cause some damage to the patient, and in this case, the truth would have to be communicated through a legal representative. Thus, doctors have a legal duty to break bad news to patients and their families.^1^

The term “bad news” means any information that is given to patients and their families, which directly or indirectly reveals any negative or severe disorder that could change their future perspectives and vision of life.^2^^,^^3^^,^^4^^,^^5^^,^^6^ Many difficulties that doctors have in breaking bad news can be explained by their fear of causing harm and suffering to their patients, and fear of being blamed for or having to deal with their patients’ emotions. All of these emotions may be unpredictable and unexpected.^7^ They may consist of denial, deep distress, blame or fear of emotions, diseases and death.^4^^,^^5^ Although some studies have stated that patients want honesty, compassion, care and affectivity, and to have their doubts clarified by their doctors,^3^ they also expect not only professionalism and competence in clinical skills, but also effectiveness of communication.^8^ However, this should not be done in a cold or careless manner.^9^ Thus, breaking bad news is difficult, unpleasant and uncomfortable. Nevertheless, it is truly necessary and requires skill on the part of healthcare professionals.^10^

To improve such skills, guidance on how to systematize breaking bad news and make it less traumatic has been provided.^2^ One example of such techniques is the SPIKES protocol, which describes six steps of communication.^2^^,^^6^^,^^11^ The first step “S”, or setting up, refers to preparation of the medical environment. The place where such news is given should preferably be private, reserved and welcoming. This is the right moment to build a good doctor-patient relationship. The second step, “P”, perception, is the opportunity to discover what the patient knows about his or her condition or disease, through open questions. The third step “I”, invitation, is the moment to analyze how much the patient wants to know, and whether he or she has any doubts to be clarified. The fourth step “K”, knowledge, is the time when everything about the diagnosis will be announced. At this moment, it is important to use simple words, without technical terms, in order to transmit the information. It is recommended that the matter should be introduced with some phrases that indicate that bad news will be transmitted. The fifth step “E”, emotions, is the time to express empathy, identify the patients’ emotions and give support. The last but not least important step “S”, strategy and summary, is the time to suggest what the treatment should be, and what the prognosis is, and also to summarize everything that has been said, in order to check that patient has understood it.^2^^,^^3^^,^^4^^,^^5^^,^^6^

Studies have shown that doctors and healthcare professionals who take a training course on breaking bad news do not depend exclusively on their own experiences or observations, and they feel more comfortable and confident when communicating such information. Thus, holding workshops that teach techniques for breaking bad news will produce better prepared and more confident professionals.^9^

## OBJECTIVE

To analyze doctors’ skills and difficulties in breaking bad news, to ascertain which specialties are better prepared for this and to assess the importance of introducing this topic to undergraduate medical students.

## METHODS

This was an observational and quantitative study conducted during 2015 and at the beginning of 2016, at a university hospital in Belo Horizonte, Minas Gerais, Brazil. The inclusion criterion for the subjects was that they should be doctors working in any sector of the university hospital. The following were excluded: doctors who did not have any contact with patients (radiologists, pathologists and laboratory workers), and those who did not sign the free and informed consent statement. The potential sample comprised the entire clinical staff of the hospital, including both residents and more senior doctors.

The research instrument used was a questionnaire structured into two parts (Annex 1). The first part consisted of five personal questions (the professional’s full name, age, specialty, work sector and length of time since graduation). The second part consisted of 17 questions on bad news concepts, medical difficulties and emotions, the importance of including breaking bad news in undergraduate courses and how doctors should break bad news. Each question was based on the SPIKES protocol and on questionnaires in other studies that were based on the same protocol. This questionnaire has been internationally validated in Spanish, but Portuguese and English versions have been used in other studies.^8^^,^^11^^,^^12^^,^^13^ All doctors answered the questionnaire and signed the consent statement within a 15-minute period at the hospital during their work time.

The hospital director signed a statement agreeing to the research. The data were gathered after the project had been approved by the research ethics committee at the School of Medical Sciences of Minas Gerais (Faculdade de Ciências Médicas de Minas Gerais, FCMMG).

The data were analyzed by means of the Epi-Info 7.1.2.0 software (2012) during October 2015 and February 2016, using frequencies, percentages and the chi-square test with significance of 0.05.

## RESULTS

An up-to-date list provided by the university hospital showed that the clinical staff totaled 160 people, consisting of 42 residents and 108 more senior doctors. Out of this total, 121 professionals provided responses for the survey (75% of the clinical staff). The remaining 25% did not participate for a variety of reasons: no direct contact with patients; unwillingness to participate; time mismatch between researcher and professional; vacation time for some professionals; or, in the view of some of the subjects, they were not responsible for delivering bad news. Table 1 shows the distribution of the participating professionals according to their specialty.

**Table 1.**
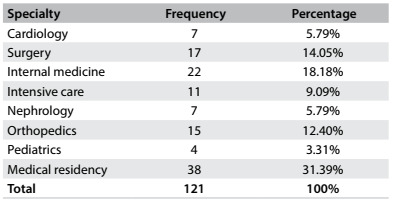
Distribution of the medical professionals analyzed, according to their specialty

Among the residents, the specialties that they belonged to were: internal medicine, surgery, anesthesiology and pediatrics. The sectors in which most of the professionals worked were surgical and intensive care units. The professionals interviewed were mostly aged 25 to 40 years (82%), with almost equal numbers of men and women.

In relation to the frequency with which these professionals gave bad news, the responses were uniform. Thus, most of them frequently (37.19%) or occasionally (28.10%) provided bad news. Moreover, most participants considered that their ability to communicate bad news was good or acceptable.

In the question on the concept of bad news, only one participant diverged from the opinion of the others. This individual believed that bad news was any information that caused physical harm to the patient. On the other hand, the response indicated by 99.17% of the participants was that bad news was any information that was transmitted with the implication of some serious negative change that could affect the individual’s outlook on life or his/her prospects for the future.

Regarding questions relating to ideas proposed through the SPIKES protocol, we obtained the following patterns. Overall, 84.3% of the participants used both verbal and non-verbal language to deliver bad news. Among the participants who had graduated less than 10 years earlier, 75% used both types of language; among those who had graduated 11 to 20 years earlier, 78%; and among those who had graduated more than 20 years earlier, 100% (P = 0.64). Both types of language were used by only 64.71% of the surgeons, but by more than 77% of the other professionals. Comparing cardiology, nephrology and pediatrics (100%) and internal medicine (95.45%) with the other specialties, use of both types of language within these four specialties was much greater (P = 0.09).

Most of the professionals (about 78%) sought a cozy private place in which to provide the news. The medical specialties of those who looked for a private place to talk were distributed as shown in Table 2. Thus, nephrologists, surgeons and residents were better prepared regarding this topic than were professionals in other specialties (P = 0.0003).

**Table 2.**
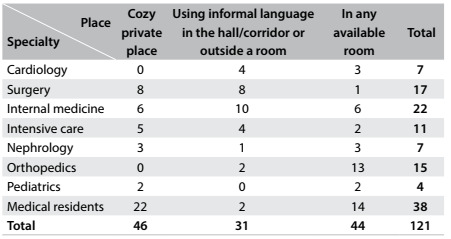
Relationship between specialty and the place where bad news is given

Concerning length of time since graduation and the demand for privacy, most of the professionals with more than 20 years of experience sought a cozy private place (83.33%). On the other hand, those with less than 10 years of experience gave bad news to their patients more often in any available room (39.56%) than in a cozy private place (31.87%). This result was statistically significant (P = 0.02).

In relation to bedridden patients, 94.12% of the professionals provided bad news while standing next to the patient’s bed. In relation to how professionals gave bad news, most of them spoke comprehensibly and clearly, while avoiding technical jargon, and they clarified doubts (Table 3).

**Table 3.**
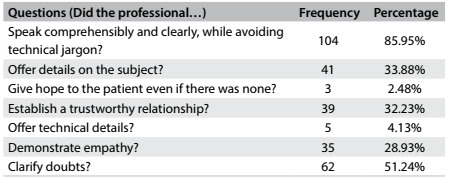
How the professionals inform patients about bad news

93.39% of the participants provided information cautiously, according to the demands of the patients and/or their relatives, and they mostly (62.29%) told the truth first to the family and then to the patient. This was also seen with regard to the length of time since graduation: the majority also told the family first and then the patient.

Concerning the skills used when the professionals broke bad news, most of them always reserved a period for clarifying doubts (51.24%), and they listened carefully without interruptions (56.20%). Regarding the length of time since graduation, those who had graduated more than 20 years earlier were open to questions and to clarification of doubts in 83.33% of the cases, which was not seen among those who had graduated 11 to 20 years or 1 to 10 years earlier. Among the medical specialties, the ones in which significantly greater time was devoted to questions were cardiology and pediatrics (100% of the professionals) and surgery (88.24%).

Concerning exploration of what patients already knew about their condition, what they wanted to know and what their concerns were, more than 53% of the doctors took an approach of this nature among their patients.

Among the specialties, the professionals who most explored what patients already knew about their health condition were: surgeons (88.24%), general practitioners (77.27%), intensivists (100%) and pediatricians (75%). In relation to what patients wanted to know, 100% of cardiologists and 100% of pediatricians discussed this. Patients’ concerns were explored by 72.73% of intensivists, 85.71% of nephrologists, 80% of orthopedists and 70.59% of first-year residents. With regard to the length of time since graduation, those with more than 10 years of experience explored these three areas most (Table 4).

**Table 4.**
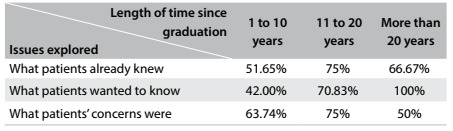
Relationship between length of time since graduation and issues explored during the conversation

Regarding the doctors’ opinions about their fears and feelings, most of them felt sad (40.83%) when they had to break bad news. They were also afraid of being blamed (66.9%) and afraid of the patients’ reactions (58.6%).

None of the participants was aware of any instrument or protocol that could help in addressing bad news and giving it to patients. Most of them had learned by watching other specialists (42.15%). The vast majority believed that adding the subject to undergraduate courses was important (45.45%) or very important (42.15%).

## DISCUSSION

All patients, to a greater or lesser degree, are distressed when sick, because of the uncertainties about their real condition and prognosis. Therefore, doctors have a duty to contribute towards relieving this anguish and not to increase it through negative gestures or expressions (iatrogenesis). Consequently, doctors need to be prudent when communicating bad news, through using appropriate skills in order to avoid inappropriate non-verbal language.^13^ In the context of non-verbal reactions, doctors’ physical appearance, use of white clothes or a white coat and attentiveness to behavioral attitude positively influence the degree of confidence that patients have in their doctor.^14^ Attitudes like making eye contact, touching hands or smiling, and expressing an empathic silence assure patients that they can count on the doctor during their period of distress. On the other hand, doctors should be prepared through adequate training and choosing apt skills, to be able to break bad news appropriately.^15^

Most of the professionals surveyed in the present study had graduated between one and ten years earlier. This high number of physicians who graduated recently may be due to the increase in the supply of undergraduate and postgraduate places at medical schools in Brazil.^11^ This is indeed a limitation of this study: it was a cross-sectional survey; the number of doctors included who had graduated more than 20 years earlier was small; and the numbers of doctors in some specialties were also small. These factors made comparisons difficult. Moreover, another important limitation was the fact that the questionnaire asked for the name of the professional, thus reducing anonymity, which may have made the responses less trustworthy.

This study revealed that these professionals “usually” or “almost always” were the ones to transmit bad news (60% of the cases). This was similar to what was found by Lench, who observed this in 65% of the cases.^11^ Regarding the concept of bad news, there was no significant divergence among the participants of the present study.

The SPIKES protocol envisages six steps in reporting bad news. The first step is the setting (S), i.e. the scenario, and this refers to doctors’ preparation and the space within which they will deliver the news. The protocol states that a private place in which there will not be any interruptions should preferably be chosen. Moreover, it needs to be ascertained whether the patient wants to be accompanied or not during the conversation. The doctor should preferably be seated next to the patient, in order to offer him/her comfort and safety, as well as showing that both are at the same level and in the same situation. It is a time to appear calm and serene and to give the patient a moment of silence to listen.^16^ These items can be analyzed through some questions in the questionnaire relating to how to give bad news (seeking a cozy private place, informing the patient while sitting at his/her bedside and listening carefully to the patient without interruptions). Most of the clinical staff in the present study said that they looked for a private environment (a specific room or an available office) in which to report the bad news, and a minority was also concerned with the comfort and coziness of the space. These findings are similar to the data of the study by Martín Hernández and Trujillo Matienzo, in which the majority of the participants also worried about privacy.^13^ Regarding the length of time since graduation, consistency with regard to seeking privacy was found. However, regarding quality, those who had graduated longer ago were more concerned about this, which could be explained by their greater experience.

The second step of the protocol is perception (P). This is the moment to check what the patient is aware of regarding his/her state of illness or condition, and to discover whether he/she would like to be informed about the condition and what his/her concerns are.^16^ Questions relating to the issues explored in the conversation need to be analyzed. It was found in the present study that around half of the professionals did not address all of the issues, thus showing that there was a deficiency in the dialogue between doctors and patients. This contrasts with a study conducted in Cuba in 2009, in which these issues were better approached and explored by the doctors (around 72%).^13^ This might be explained by lack of preparation among the professionals in our institution. It may also have been because the professionals’ fears were mostly based on the patients’ reactions and the possibility of taking away their hopes. Regarding the issues explored, none of the specialties was considered to be different from the others. Nevertheless, in relation to the length of time since graduation, these issues were covered more by the professionals who graduated more than 20 years earlier, which suggests that experience is essential in approaching patients regarding their health conditions.

The third step is the invitation (I). This is the time at which the degree of knowledge that patients want to have regarding their condition needs to be ascertained, while leaving time for the patient and/or family to ask questions.^16^ At this point, the percentage of physicians who provided this opening for dialogue with their patients was analyzed. Only half of the professionals (among whom most had graduated more than 20 years earlier), confirmed that they offered this moment to their patients. This finding was not too different from what was reported in the study by Martín Hernández and Trujillo Matienzo, who found that this moment was offered only by 69% of the professionals.^13^ In conclusion, a failing among the clinical staff was clearly noticeable, given that this attitude is important for the purpose of this step. However, the doctors may have had difficulty in implementing this because of their own fears regarding giving bad news. At this point, another deficiency among most doctors can be observed, given that they do not offer this essential moment to their patients, even though receiving bad news is a moment of anguish and discomfort.^16^

The fourth step, of knowledge (K), is the moment before and during communication of bad news. At this moment, the patient has been prepared and is expecting that bad news will be announced. Clear verbal and non-verbal language needs to be used, and technical jargon needs to be avoided. The information needs to be given gradually, so as to make sure that the patient understood everything.^16^ In the present study, 84.3% of the doctors used both verbal and non-verbal language to talk with their patients. These results were far superior to the ones found in a study conducted in 2009, in which this proportion was only 40.9%.^13^ However, it was reported in that study that this occurred because many of the professionals did not consider gestures and facial expressions to be non-verbal language, or were not even aware that they were using it. To avoid this bias during the interviews that we conducted in our university hospital, we explained and exemplified what non-verbal language would consist of. In relation to the length of time since graduation, there was much divergence, although most of the doctors used both verbal and non-verbal language. The majority of the professionals informed their patients according to their demands or those of family members. There was a discrepancy in relation to a study conducted in Cuba, in which despite the authors’ opinion that the communication should be performed in a slow, gradual and continuous manner, 41.8% of the professionals did not do this. In relation to use of clear, understandable language and detailed explanations, the present study corroborates the findings from Cuba, given that the percentages were similar (such that clear and understandable language was used in 85.95% and 95.9% of the cases respectively and detailed explanations in 33.88% and 33.7% respectively).^13^ In this regard, both of these studies endorse this step of the protocol, and differ only in details, which need to be better addressed by both studies.

The fifth step, of empathy (E), is the moment for doctors to show their patients that they have established a relationship of trust with them, and that they understand their patients’ feelings and are compassionate to the situation.^16^ Meanwhile, doctors should not take away patients’ hopes, or feed them false hopes, although some professionals were seen to have done this. This is the moment when doctors should show their patients support in several ways, especially emotionally and spiritually. After all, when patients and their families receive bad news, this arouses feelings, emotions and concerns. Regarding emotional and spiritual support, the data from the present study resembled the findings of the 2009 study (90.08% and 74.38%), thus showing that support was provided for both patients and their families.^13^ However, a very small number of the professionals in the present study provided false hope for their patients. These doctors had all graduated less than 10 years earlier. About one-third of the professionals established a relationship of trust with their patients and displayed empathy. In relation to establishing a relationship, the data differed completely from the findings in Cuba, where more than half of the doctors established a relationship of trust with their patients. On the other hand, in relation to empathy, the data were similar.^13^ This may have occurred through lack of understanding of the term “put yourself in the patient’s shoes”, which required the doctor to be empathetic and understand the patient’s feelings and reactions. It could even be explained as reported by the author of the 2009 study, i.e. that offering trust and empathy to patients is not an easy task, even though it is important.^13^ After all, giving bad news is a stressful situation for both patient and doctor, and therefore those who can avoid it always will.^8^

The sixth and final step is strategy and summary (S). This is the moment to ascertain whether the patient has understood all the information and perform a brief retrospective analysis, as well as presenting and discussing a therapeutic plan and prognosis for the illness, with the patient.^16^ Therefore, as in the 2009 study, the participants preferred to talk firstly to the family and afterwards to the patient. Independent of the length of time since graduation, the majority of the professionals adopted this position. Consequently, it can be inferred that doctors are not prepared to talk about prognoses and treatments with their patients, since they delegate an attribution that should be part of the doctor-patient relationship, to family members.^13^

None of the participants was aware of any instrument or protocol that could help in addressing patients when bad news needs to be communicated. This result corroborates the findings of Lench and Destefani, who showed that 60% of the participants in their study were unaware of the SPIKES protocol. The longer the time since graduation was, the less was known about this subject.^11^ Thus, the participants had learned through observing other specialists. The vast majority believed that adding the subject of how to give bad news to the undergraduate curriculum was important or very important, given that as shown by Lench, 69% of the participants had had no training on this subject and 60% considered that they had had a poor learning experience on this subject.^11^ Therefore, the skills relating to giving bad news need to be improved. This medical skill can be improved through using a protocol as a guide for transmitting bad news, as well as through inclusion of “how to give bad news” in undergraduate courses. One proposed approach for such inclusion consists of teaching communication skills through dramatization and studying medical ethics and bioethics.^17^^,^^18^

## FINAL REMARKS

From the results obtained, it can be concluded that many doctors have not developed sufficient skills relating to conveying bad news, since the crucial basic points of empathy and a good relationship of trust between doctors and their patients have not been well explored and worked out. In addition, professionals seem to be afraid to address certain issues among their patients (i.e. what patients already know and what they want to know about their health conditions). However, emotional, spiritual, informational and instrumental support is generally provided to both patients and their families. This is very important, given that bad news not only affects patients, but also their families.

In relation to the specialties, no conclusion can be reached regarding which of them are best prepared for the task of breaking bad news. However, regarding the length of time since graduation, we can conclude that in general, doctors with more than 10 years of experience since graduation, and especially those with more than 20 years of experience since graduation, were better qualified to provide bad news.

## CONCLUSIONS

Methods for improving communication of bad news have been proposed and may be applied in medical practice in order to complement the course and improve medical skills.
